# Lassa fever in pregnancy: a systematic review and meta-analysis

**DOI:** 10.1093/trstmh/traa011

**Published:** 2020-03-03

**Authors:** Nzelle D Kayem, Charlotte Benson, Christina Y L Aye, Sarah Barker, Mariana Tome, Stephen Kennedy, Proochista Ariana, Peter Horby

**Affiliations:** 1 Nuffield Department of Medicine, University of Oxford, Oxford, OX3 7LG, UK; 2 Women's Centre, John Radcliffe Hospital, Oxford University Hospitals, Oxford, OX3 9DU, UK; 3 Nuffield Department of Women's & Reproductive Health, University of Oxford, Oxford, OX3 9DU, UK; 4 Stoke Mandeville Hospital, Mandeville Road, Aylesbury, HP21 8AL, UK

**Keywords:** case fatality rate, Lassa fever, perinatal mortality, pregnancy outcomes

## Abstract

Lassa fever is a zoonotic infection endemic to West Africa and is known to have adverse effects in pregnancy. We sought to synthesize and critically appraise currently available evidence on the effects of Lassa fever in pregnancy. An exhaustive bibliographic search from dates of inception to 30 September 2019 yielded 13 studies, from which individual patient data were extracted. The absolute risk of maternal death associated with Lassa fever was estimated at 33.73% (95% CI 22.05 to 46.42%, I^2^=72.40%, p=0.0014). The relative risk of death in pregnant women compared with non-pregnant women was estimated at 2·86 (95% CI 1.77 to 4.63, I^2^=27.27%, p=0.239). The formal gap analysis shows imprecise data on the risk of Lassa-related maternal and perinatal mortality and insufficient data for other pregnancy outcomes. The currently available evidence for the use of ribavirin in pregnant patients is not conclusive. With a threefold increased risk of mortality, there is a need to prioritize pregnant women as a special subgroup of interest for Lassa research. Robust prospective studies estimating the true incidence of adverse maternal and perinatal outcomes and randomized controlled trials to evaluate the efficacy of therapeutics for maternal Lassa virus infection are urgently needed.

## Introduction

Lassa fever is a zoonotic infection endemic to West Africa. Most infections are asymptomatic with estimates of over 300 000 infections occurring in the region each year.^1^^,^^2^ In the general population, mortality rates range from 1–2% in mild cases^2^^,^^3^ to 15–20% in severe cases.^3–5^ The crude case fatality rate in confirmed cases, in the 2019 Lassa fever outbreak in Nigeria, was 21.4%.^6^ Reports of Lassa fever in pregnant women indicate a poorer prognosis with maternal case fatality rates ranging from 7% in early pregnancy to 30% in late pregnancy.^7^ Neonatal and fetal losses are reportedly high at 75 and 92%, respectively, with most fetal losses occurring in early pregnancy.^7^

We sought to summarize, synthesize and critically appraise currently available evidence from peer-reviewed and gray literature on the effects of Lassa fever in pregnancy. An understanding of the disease epidemiology in different population groups, particularly in vulnerable groups, facilitates prioritization of research and control strategies. We highlight where the current limitations to the evidence lie and discuss avenues for further research that may inform the Lassa fever research agenda and facilitate the development of preventative and curative measures for Lassa fever with pregnant women as a special subgroup of interest. We look specifically at the clinical characteristics, the maternal and perinatal outcomes of Lassa fever during pregnancy and the clinical management practices for maternal Lassa virus infection. 

## Materials and methods

### Search strategy and selection criteria

The systematic review and meta-analysis were conducted as part of a broader review looking at five priority viral hemorrhagic fevers listed on the WHO’s research and development blueprint.^8^ These include Ebola virus disease, Marburg virus disease, Lassa fever, Rift Valley fever and Crimean-Congo hemorrhagic fever. We followed the Preferred Reporting Items for Systematic reviews and Meta-Analysis (PRISMA) guidelines for conducting systematic reviews.^9^

**Figure 1:**
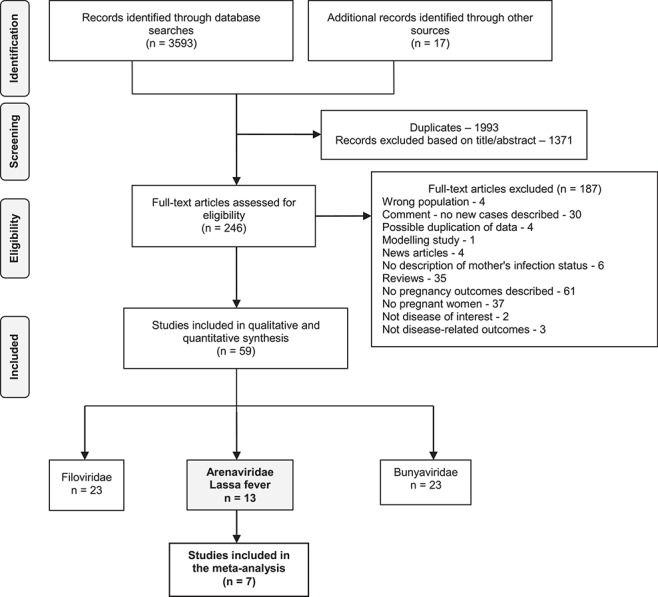
PRISMA flow diagram of selected studies. n, number of papers.

The methods are explained in detail in the Supplementary data. Briefly, we searched the following bibliographic databases from their respective inception dates until 25 June 2018, with an updated search on 30 September 2019: PubMed, Web of Science, Excerpta Medica database (EMBASE), the WHO Global Health Library (WHOGL) and Cumulative Index to Nursing and Allied Health Literature (CINAHL).

Additionally, clinical trial databases such as the Cochrane Central Register of Controlled Trials, the Cochrane Pregnancy and Childbirth Register, the Cochrane Infectious Diseases Register, the International Standard Randomized Controlled Trial Number (ISRCTN) registry, the WHO International Clinical Trials Registry Platform, the European Union Clinical Trials Register, the Pan African Clinical Trials Registry and the ClinicalTrials.gov database were searched. The references from relevant reviews and included studies were also searched for additional citations.

The search strategy used a combination of Medical Subject Headings (MeSH terms) and keywords to capture pregnancy and the selected viral hemorrhagic fevers (Supplementary Table 1 shows some of the search terms used). All study designs were considered for inclusion. There were no language or date restrictions. A standardized, prepiloted form was used to extract data from the included studies.

### Data analysis

The citations were screened using an online systematic review software program: Rayyan.^10^ For each citation, two reviewers independently screened the titles and abstracts and assessed the full texts for eligibility. Data were independently extracted and any discrepancies were resolved through discussion with a third author.

We extracted dichotomous data from the studies to generate proportions or ORs depending on the available information, irrespective of the study quality. We only performed a meta-analysis when two or more studies reported on the same outcome and included at least five pregnant women or live births. The gestational age at which outcomes occurred was not reported in most studies; hence, all deaths occurring before or during labor and delivery were grouped as fetal loss.

The three most commonly reported outcomes (maternal death, fetal loss and neonatal death) were summarized in meta-analysis forest plots. Where the information was available, we calculated the ORs by comparing the odds of death in pregnant Lassa-positive women with non-pregnant Lassa-positive women.

Two authors independently assessed the risk of bias in the included studies, using pre-existing tools appropriate to the study design. Any discordance was resolved by consensus or discussion with a third reviewer. We used a tool developed by Murad et al.^11^ to assess the quality of case reports and case series studies, and the modified Newcastle-Ottawa scale^12^ to evaluate the risk of bias in cohort-type studies (Supplementary Table 2a-c). We provide aggregated quality scores for each citation but the quality scores were also color-coded to allow for a better interpretation of the study quality by readers, given that an aggregated score fails to highlight where weaknesses in the reported study design are found.^13^

All statistical analyses were performed using R statistical software version 3.6.1.^14^ We used the *metaprop* command for the proportional meta-analysis because it implements the Freeman-Tukey double arcsine transformation,^15^^,^^16^ which is well-suited to binomial data with extreme proportions and stabilizes variances in proportions.^15^^,^^16^ A random effects model was used to calculate a weighted summary estimate and the 95% CI for the proportion of maternal deaths, fetal loss or neonatal deaths in Lassa fever. We estimated the following parameters: Cochran's Q and its associated p-value, tau squared (τ)^2^ and Higgins I^2^. For our study, the degree of heterogeneity was interpreted as none (<25%), low (25–49%), moderate (50–74%) or high (≥75%).^17^ Where I^2^ was high, we evaluated reasons for the observed variances.

Sensitivity analysis was performed post-hoc by excluding studies with 0 or 100% proportion and studies with small sample sizes (<10 pregnant women). Our aim was to assess if excluding studies with smaller sample sizes and with extreme proportions would result in a statistically significant difference in summary estimates. A meta-regression was performed to assess the effect of the study design (cohort or other design), the sample size (less than or greater than 10 pregnant women) and the year in which the outbreak occurred (before or after 2000) on the summary estimates. Peter's test was used in combination with a funnel plot to assess potential publication bias.^18^

A formal analysis using a framework developed by Robinson et al.^19^ was used to identify research gaps. The framework was modified to have five categories (Supplementary Table 3a) by separating option A in Robinson et al.'s framework^19^ (insufficient and imprecise information) into two distinct categories, A for insufficient data and B for imprecise data. For each objective, we presumed certain outcomes should be reported (Supplementary Table 3b) and as such assessed the evidence or lack thereof based on these objectives. We registered this review in PROSPERO, the international prospective register of systematic reviews of the University of York and the National Institute for Health Research, under protocol number CRD42018097022.

## Results

An initial search of systematic review databases showed that there was no systematic review on the effects of Lassa fever during pregnancy. We identified a total of 3610 records and excluded 1371 due to lack of primary data and inapplicability to the review objectives (Figure 1). Two hundred and forty-six full texts were assessed for eligibility, of which 59 studies met the inclusion criteria. Thirteen studies included pregnant women with Lassa virus infection,^7^^,^^20–31^ and seven of these studies were included in the meta-analysis. There were a total of 276 pregnant women included in the studies. The characteristics of the included studies are summarized in Table 1. The age of pregnant women was reported in 41 of 276 pregnant women and ranged from 16 to 39 y.^20^^,^^21^^,^^24^^,^^30^^,^^31^ We could not ascertain the mean or median age of presentation because such data were not provided. Similarly, individual data on gestational ages at presentation or related to outcome were inconsistently reported and ranged from 5 wk to term (≥36 wk) in 110 pregnant women.^7^^,^^20–22^^,^^24^^,^^30^^,^^31^

### Clinical characteristics and course of maternal Lassa virus infection

The clinical features of maternal Lassa fever were generally non-specific and were recorded in 103 of 276 pregnant women in six studies,^7^^,^^20–22^^,^^30^^,^^31^ but only two studies satisfied the criteria for a meta-analysis. The frequency of symptoms and resulting complications reported in five or more pregnant women are narratively summarized in Table 2. We present aggregated proportions for the symptoms with corresponding weighted summary proportions where these could be estimated.

The length of hospital stay ranged from 2–18 d reported in nine pregnant women,^21–23^^,^^31^ while the time between illness onset and admission ranged from 2–14 d in 33 pregnant women,^21^^,^^30^^,^^31^ and the mean time from illness onset to treatment was 3–14 d, reported in 33 pregnant women.^21^^,^^30^^,^^31^

### Maternal outcomes of Lassa fever during pregnancy

**Figure 2:**
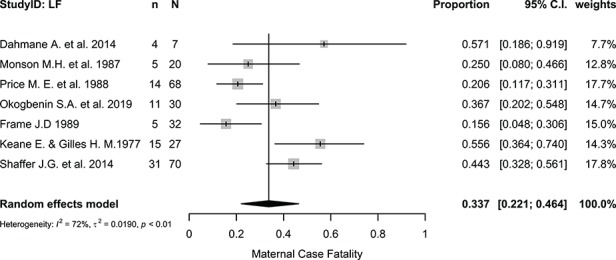
Proportional meta-analysis forest plot of studies reporting maternal death for Lassa virus infection in pregnancy. I^2^, Higgins statistic, τ^2^, tau squared, p, p-value associated with Cochran's Q for heterogeneity; LF, Lassa fever; n, number of maternal deaths; N, total number of pregnant women included in the analysis.

Maternal death was the most commonly reported maternal outcome with a pooled case-fatality proportion of 33.73% (95% CI 22.05 to 46.42%, I^2^=72.40%, p=0.0014; Figure 2). Gestational ages at which maternal mortality occurred were not reported; therefore, an analysis of maternal death by trimester could not be performed.

**Figure 3:**
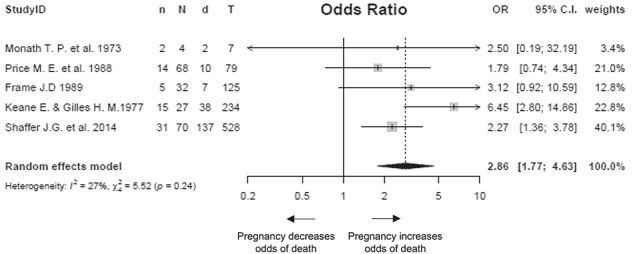
Forest plot showing the risk of death from Lassa fever in pregnant women compared with non-pregnant women. I^2^, Higgins statistic, τ^2^, tau squared; p, p-value associated with Cochran's Q for heterogeneity; LF, Lassa fever; n, number of maternal deaths; N, total number of pregnant women included in the analysis; d, number of deaths in non-pregnant women; T, total number of non-pregnant women included in the analysis.

Five studies reported on maternal death as well as death in the non-pregnant population.^7^^,^^22^^,^^25^^,^^28^^,^^29^ We combined these studies in a meta-analysis to assess the odds of death in pregnant women with Lassa fever compared with non-pregnant women with Lassa fever. The pooled OR was 2·86 (95% CI 1.77 to 4.63, I^2^=27.27%, p=0·239; Figure 3). We could not compare the outcomes between Lassa-positive pregnant women with Lassa-negative pregnant women because of a lack of adequate data.

**Table 1:** Characteristics of the studies included in the review

Study ID and country	Enrolment period	Study design	Cases - definition	Controls - definition	Number of pregnant women in the study	Status of cases diagnostic tests used (viral strain)	Gestational age estimation methods	Methods used to correct for confounding
Agboeze et al., 2019; Nigeria	Mar 2019	Case report	1 pregnant woman with confirmed laboratory diagnosis of LF	NA	1	Confirmed PCR	LNMP	NA
Okogbenin et al., 2019; Nigeria	Jan 2009 to Mar 2018	Cohort retrospective	Laboratory-confirmed LF with data available for signs and symptoms (only pregnant women)	NS	30	Confirmed PCR	LNMP and/or USS	NS
Bello et al., 2016; Nigeria	16 Sep 2014 to 2 Oct 2014	Case series retrospective	2 pregnant women with LF	NA	2	Confirmed PCR, ELISA-Ag, IgM and IgG	USS	NA
Dahmane et al., 2014; Sierra Leone	Apr 2011 to Feb 2012	Cohort retrospective	84 patients admitted with suspected LF from April 2011 to February 2012 (73 children, 10 pregnant, 1 male)	NS	10 (7 confirmed)	Confirmed, suspected ELISA IgM and IgG	NS	NS
Shaffer et al., 2014; Sierra Leone	2008 to 2012	Cohort retrospective	1740 all patients from Sierra Leone suspected with LF who had samples sent to Kenema, of which 595 were confirmed Ag-positive and/or IgM- positive	Cohorts of suspected or confirmed LF- positive non-pregnant women (528) and patients who were antigen and antibody IgM-negative but IgG-positive or -negative (1141)	70	Confirmed ELISA Ag and/or IgM	Self-reported	Logistic regression
Branco et al., 2011; Sierra Leone	20 Jan 2011	Case report retrospective	1 pregnant woman who traveled to LF risk area and presented with symptoms	NA	1	Confirmed PCR, ELISA Ag (LASV Macenta Z158 strain identified)	Fundal height	NA
Okogbenin et al., 2010^*^; Nigeria	Feb 2008 to Aug 2009	Case series retrospective	7 cases of PCR-confirmed LF in pregnancy managed at the Irrua Specialist Teaching Hospital, Irrua, Nigeria	NA	7	Confirmed PCR	NS	NA
Ehichioya et al., 2010; Nigeria	Feb 2005 to Mar 2008	Case series retrospective	10 patients involved in a nosocomial outbreak	NA	1	Confirmed PCR, ELISA, IgM and IgG	NS	NA
Frame 1989; Liberia	Jul 1980 to Apr 1986	Cohort retrospective	253 suspected LF cases with hemorrhagic manifestations at Curran Lutheran hospital	NS	32	Confirmed, probable and suspected IFA, virus isolation (culture) (LASV Josiah strain identified)	NS	NS
Price et al., 1988; Sierra Leone	1981 to 1985	Cohort prospective	68 women diagnosed with LF who were admitted to the study hospital and were pregnant	79 non-pregnant LF-positive admitted to the same hospital	68	Confirmed ELISA IgG and IgM, virus isolation (culture)	Fundal height	NS
Monson et al., 1987; Liberia	Jan 1980 to Mar 1984	Case series prospective	33 pediatric LF with 20 fetal or congenital LF when mother was positive for LF and children with suspected/confirmed congenital LF	NA	20	Confirmed, suspected, probable IFA, virus isolation - culture	NS	NA
Keane & Gilles, 1977; Sierra Leone	Jan 1973 to Mar 1976	Cohort retrospective	264 Patients admitted to Panguma Hospital from Jan 1973 to March 1976 with a diagnosis of LF and 108 from Segbwema in 1975	NS	30	Confirmed, suspected, probable complement fixation	NS	NS
Monath et al., 1973; Liberia	2 Mar 1972 to 6 Apr 1972	Case series retrospective	11 cases admitted to Curran Lutheran hospital between March and April 1972, with positive exposure to a young pregnant patient admitted for threatened abortion	NA	4	Confirmed, suspected virus isolation - culture, complement fixation	NS	NA

Abbreviations: IAg, antigen; IFA, immunofluorescence assay; LF, Lassa fever; LASV, Lassa virus; NA, not applicable; NS, not stated; USS, ultrasound; LNMP, last normal menstrual period.

^*^This study^24^ has been excluded from all meta-analyses because there is the risk of duplication of some of its results in another study^30^ already included, given that their enrolment periods overlap.

**Table 2:** Clinical characteristics of pregnant women with Lassa fever

Clinical feature/complication	n	N	Aggregated proportion (%)	Weighted summary proportion (%) [95% CI]
Nausea/vomiting^21^^,^^22^^,^^30^	21	33	63.64	ND
Headache^22^^,^^30^	17	31	54.84	ND
Fever^7^^,^^20–22^^,^^30^^,^^31^	54	103	52.43	72.9 [0.4 to 100%], I^2^=98.6%
Breast pain^30^	13	30	43.33	ND
Abdominal pain^20–22^^,^^30^^,^^31^	14	35	40.00	ND
Difficulty swallowing^30^	12	30	40.00	ND
Retrosternal pain^7^^,^^30^	38	98	38.78	44.53 [13.2 to 78.4%], I^2^=90.6%
Overt bleeding unspecified^20^^,^^22^^,^^30^^,^^31^	12	33	36.36	ND
Cough^20^^,^^30^	11	31	35.48	ND
Vaginal bleeding^7^^,^^30^^,^^31^	35	99	35.35	36.50 [20.7 to 53.9%], I^2^=62.0%
Pharyngitis^7^^,^^30^	30	98	30.61	30.46 [21.6 to 40.1%], I^2^=0.0%
Renal angle tenderness^30^	9	30	30.00	ND
Conjunctivitis^7^	19	68	27.94	ND
Seizures^30^	8	30	26.67	ND
Oliguria^30^	8	30	26.67	ND
Jaundice^21^^,^^30^	8	32	25.00	ND
Preterm labor^7^^,^^30^^,^^31^	15	89	16.85	15.98 [2.5 to 36.9%], I^2^=79.3%
Bilateral deafness^30^	5	30	16.67	ND
Puerperal sepsis^7^	6	56	10.71	ND

The table itemizes the reported clinical features and complications of Lassa fever occurring in pregnant women in order of decreasing frequency.

n, number of pregnant women who presented with a symptom; N, total number of pregnant women in whom a particular symptom was assessed; I^2^, Higgins statistic; ND, not done because only one study qualified for meta-analysis. Oliguria is defined as <0.5 mL/kg/h for ≥6 h

### Perinatal outcomes of maternal Lassa virus infection

**Figure 4:**
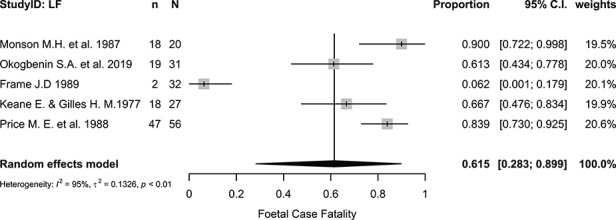
Proportional meta-analysis of studies reporting fetal loss from Lassa fever in pregnancy. I^2^, Higgins statistic, τ^2^, tau squared, p, p-value associated with Cochran's Q for heterogeneity; LF, Lassa fever; n, number of fetal losses; N, total number of fetuses included in the analysis.

**Figure 5:**
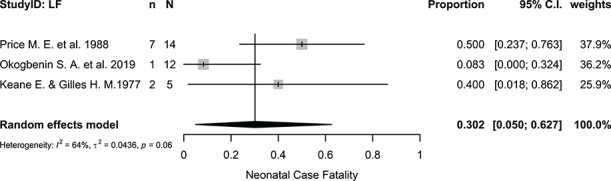
Proportional meta-analysis of neonatal death from Lassa fever. I^2^, Higgins statistic; τ^2^, tau squared; p, p-value associated with Cochran's Q for heterogeneity; LF, Lassa fever; n, number of neonatal deaths; N, total number of live births included in the analysis.

The pooled fetal case-fatality proportion was 61.50% (95% CI 28.32 to 89.86%, I^2^=94.50%, p<0.0001; Figure 4), while the overall neonatal case-fatality proportion was 30.15% (95% CI 4.96 to 62.67%, I^2^=63.90%, p=0.063; Figure 5).

The age at which neonatal death occurred was reported in only five neonates and ranged from a few hours after birth to 18 d.^26^^,^^28^^,^^30^ The gestational ages at which fetal outcomes occurred were reported in 22 pregnant women in four studies,^20^^,^^21^^,^^30^^,^^31^ three of which were case reports; therefore, it was not possible to assess the risk of fetal outcomes by trimester. Other perinatal outcomes reported in the literature include prematurity^7^^,^^31^ and vertical transmission.^26^

Clinical features of suspected Lassa virus infection in neonates were reported in a total of five neonates.^22^^,^^26^ These included fever in all five,^22^^,^^26^ bleeding in one,^26^ and generalized swelling associated with abdominal distension and bleeding referred to as swollen baby syndrome was reported in three neonates.^26^

### Clinical management practices for Lassa fever in pregnancy

The antiviral ribavirin was used in seven studies,^7^^,^^20^^,^^24^^,^^27^^,^^29–31^ two case reports,^20^^,^^31^ one case series^24^ and four cohort studies.^7^^,^^27^^,^^29^^,^^30^ The number of pregnant women receiving ribavirin was only specified in four of the seven studies,^7^^,^^20^^,^^30^^,^^31^ two of which were case reports. Overall, 43 pregnant women received ribavirin; of these, 32 survived.^7^^,^^20^^,^^30^^,^^31^ The summary estimate of the proportion of pregnant women who survived while on ribavirin was 73.94% (95% CI 57.71 to 87.63%, I^2^=10.70%, p=0.29; the Supplementary Figure 1). However, there were no data available to compare the survival rates among those who received ribavirin with those who did not receive ribavirin. Similarly, there was insufficient information on the efficacy of ribavirin in non-pregnant women.

Immunotherapy was rarely used; one cohort study indicated the use of convalescent plasma (CP)^28^ but the number of pregnant women who received CP was unspecified and the outcome of the patients was not indicated.

**Figure 6:**
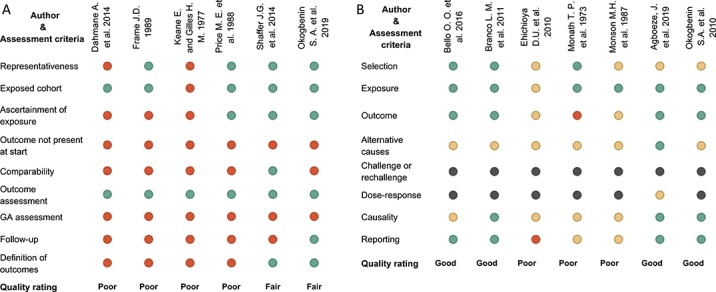
Risk of bias assessment of studies included in the systematic review and meta-analysis on Lassa fever in pregnancy. A, quality score for cohort studies; B, quality score of case reports and series. For cohort-type studies: green, star ^(*)^ and red, no star; for case series or case reports: green, yes, red, no, yellow, unclear/unsure, black, not applicable. GA, gestational age.

Obstetric management of pregnant women with Lassa fever was not recorded in most studies, thus, we were unable to evaluate if any changes in obstetric management were a result of Lassa virus infection and the efficacy of these interventions. The management of neonates born to mothers with Lassa fever was described in one study.^30^

### Sensitivity analysis and meta-regression

Post-hoc sensitivity analysis did not have a significant impact on the summary estimates obtained. Excluding studies with sample sizes of <10, the odds of maternal death became 2.91 (95% CI 1.68 to 5.05), maternal case fatality proportion was 32.192% (95% CI 20.47 to 45.09%) and neonatal case fatality proportion was 27.27% (95% CI 0.00 to 72.47%). We could not perform a sensitivity analysis for fetal losses and for clinical features because none of the studies qualified.

In the meta-regression, we assessed the overall effect of the sample size, study design and the year in which the outbreak occurred. We found that these variables did not account for the observed heterogeneity in the weighted summary estimates, p>0.1 (maternal death p=0.4260, fetal loss p=0.8820). Other potential confounders included in this review were the virus strain and the method used to confirm Lassa fever but there were insufficient data to include these variables in the model.

**Table 3:** Results of the research gap analysis

Gap criteria	Outcome (references)	Justification
A	Clinical course of infection^21–23^^,^^31^	There is little or no information on the clinical course of Lassa fever in pregnancy and it was not reported in sufficient detail for a meta-analysis.
A	Other maternal complications of Lassa virus infection^7^^,^^21^^,^^24^	A meta-analysis assessing complications of Lassa fever in pregnancy could only be conducted for preterm labor (Table 2). There is insufficient information on other complications of Lassa fever that may occur in pregnancy such as postpartum hemorrhage and other complications such as hepatitis are intermittently reported with no clear evidence on the temporality of these complications.
A	Other perinatal complications of maternal Lassa fever^7^^,^^26^^,^^31^	Prematurity was reported in seven newborns in two studies, one of which is a case report, as such, limiting further synthesis. Similarly, perinatal complications such as small-for-gestational-age and intrauterine growth restriction/retardation, low birthweight, birth defects or congenital disorders, vertical transmission were almost never assessed.
A	Definition of outcome measures	A recurrent problem during the review was the lack of definitions for outcome measures and when present usually varied from one paper to another.
A	Trimester of pregnancy and gestational age-estimation methods ^7^^,^^20–22^^,^^24^^,^^30^^,^^31^	Gestational ages were described in 110 pregnant women, however, there were not enough individual patient data to allow for an assessment of outcomes with respect to the trimester of pregnancy. The gestational age-estimation methods were not specified and it was impossible to evaluate the accuracy of the gestational ages.
A	Clinical features and complications in newborn^22^^,^^26^	Clinical features or complications in neonates born of mothers with Lassa virus infection during pregnancy were reported in a few studies but the information was insufficient to enable a meta-analysis.
A	Management of neonates ^30^^,^^31^	None of the studies describes the management of neonates born to mothers with Lassa fever.
B	Management of pregnant mothers with Lassa fever^^7^^,^^20–22^^,^^24^^,^^25^^,^^27^^,^^29–31^^,^^34^^	Different management strategies for Lassa fever in pregnancy were reported but there is insufficient information to investigate the effectiveness of different drugs or therapeutic agents. There is no information on how best to manage pregnancy and its complications in a Lassa virus-positive pregnant women or changes made to obstetric procedures due to Lassa virus infection.
B	Fetal loss^^7^^,^^20–26^^,^^30^^,^^31^^,^^34^^	The proportional meta-analysis showed a high amount of between-study heterogeneity, with wide CIs.
B	Neonatal death ^^7^^,^^22^^,^^26^^,^^30^^,^^31^^,^^34^^,^^	Although a meta-analysis was conducted, there are few studies included in the meta- analysis and the sample sizes are small. Importantly, we need a better understanding of the mechanisms by which neonatal infection occurs.
B	Maternal mortality – absolute risk^^7^^,^^21–27^^,^^29–31^^,^^34^^	We conducted a meta-analysis looking at the absolute risk of maternal death from Lassa fever and, while the estimate might be precise given the narrow CI, there is a high amount of between-study heterogeneity and our meta-analysis has <10 studies. Additionally, most of the studies in the meta-analysis are cohorts with a high risk of bias.
C	Maternal mortality – relative risk^^7^,^25–27^^,^^29^^,^^30^^,^^34^^	The relative risk of death among pregnant women (OR 2.86) has a narrow CI and low between-study heterogeneity. There is, however, a moderate-to-high risk of methodological bias among the included cohort studies.
D	Coinfections and comorbidities ^20–22^^,^^30^^,^^31^	While coinfections and/or comorbidities were reported in five studies; three of these were case reports. Among the cohort studies, coinfections were reported in one pregnant woman and eight pregnant women, respectively. None of the studies assessed the effects of different comorbidities or coinfections on the clinical course of Lassa fever in pregnancy.

The gap analysis evaluates gaps in the literature based on the expected outcomes of the review, which were the maternal and perinatal outcomes of Lassa fever, clinical management strategies and clinical features and course of maternal Lassa virus infection. The table summarizes the different gaps identified, providing references for studies which reported the outcomes and a justification for the gap described.

A, no data or insufficient information.

B, meta-analysis conducted; however, a few studies in the meta-analysis of studies included have small sample sizes, or the meta-analysis is associated with a high amount of heterogeneity and/or wide or extremely wide CIs.

C, some of the studies included in the meta-analysis have a low-quality rating (that is a moderate or high risk of methodological bias).

D, most of the studies discussing the specific outcome are case report or case series studies.

### Risk of bias assessment

The quality of the studies is displayed in Figure 6. Among the case series studies, the aggregated risk of bias ranged from low in four studies to high in three studies. Among the cohort studies, only two of six cohort studies had a moderately low risk of bias. The methodological bias in most studies was particularly significant with respect to the determination of temporality and measurement of outcomes (Figure 6).

### Publication bias

Funnel plots for publication bias showed some asymmetry (the Supplementary Figure 2). Peter's test, however, showed no evidence of publication bias, p>0.1 (maternal case fatality proportion: p=0.812, fetal case fatality proportion: p=0.593 and maternal OR for pregnant women compared with non-pregnant women: p=0.814).

### Gap analysis

A formal assessment for potential research gaps emphasized the paucity of evidence on the effects of Lassa fever in pregnancy (Table 3). While Lassa fever is believed to have severe adverse outcomes in the pregnant woman, her unborn fetus and the newborn, the actual risk of most maternal and perinatal outcomes remains unknown and the absolute risk of maternal, fetal and neonatal deaths cannot be conclusively defined. In the same way, the efficacy of different therapeutics for maternal Lassa virus infection remains unknown. These knowledge gaps are a result of a combination of factors.

First, there are insufficient data on the clinical characteristics and course of maternal Lassa virus infection and the maternal and perinatal complications associated with Lassa fever in pregnancy. Similarly, while ribavirin and convalescent plasma were reportedly used to manage pregnant women, there are insufficient data to estimate the efficacy of ribavirin or other therapeutic agents in the management of maternal Lassa virus infection. There are no studies to date that have assessed the complications associated with Lassa virus in pregnancy and the mechanisms by which these occur. Evidence synthesis was also limited by the lack of definitions for outcome measures and, when present, usually varied from one paper to another. Likewise, while gestational ages were described in 110 pregnant women, there were insufficient individual patient data to allow for an assessment of outcomes with respect to the trimester of pregnancy.

Second, where data are available, the methodological differences in the data result in imprecise estimates, with high amounts of between-study heterogeneity, and some outcomes are only discussed in descriptive studies such as case reports or case series studies.

Finally, despite the exhaustive search, there are only 13 studies that discuss pregnant women with Lassa fever reporting on one or other outcome and, of these, only two cohort studies have a fair quality score. Seven of the studies have a high risk of methodological bias, and of these four are cohort studies, which further limits the strength of the currently available evidence.

## Discussion

This systematic review and meta-analysis shows that pregnant women are almost threefold more likely to die as a result of Lassa virus infection than their non-pregnant counterparts. This estimate for the relative risk of death in pregnant women compared with non-pregnant women is statistically significant (p<0.0001) with narrow CIs (95% CI –1.77 to 4.63) and low levels of heterogeneity (I^2^=27.27%, p=0.239). The studies included in the meta-analysis had a moderate to high risk of bias; even so, this estimate is similar to that observed by Schieffelin et al. (OR=2.62).^32^ The current evidence underscores the need to prioritize pregnant women as a special group of interest.

All the weighted summary estimates for absolute risk of death are associated with high amounts of between-study heterogeneity, which are not explained by the differences in study design, sample size or year in which the outbreak occurred. We did not have sufficient data to further analyze the effect of other explanatory variables such as health system factors, different viral strains, the severity of illness and trimester of pregnancy, which may also account for the high heterogeneity observed. Robust prospective studies are urgently needed to estimate the true incidence and risk of mortality and morbidity associated with Lassa fever in pregnancy and the underlying pathophysiological mechanisms associated with maternal and perinatal outcomes.

Clinical features of Lassa fever described in the included studies were generally non-specific and are common to a wide range of other endemic infections in West Africa,^4^^,^^33^ making the diagnosis of Lassa fever a challenge. While constellations of risk factors and clinical or laboratory features may be suggestive of Lassa fever, it is unlikely that any would have a high predictive value. Point-of-care rapid diagnostics for Lassa virus and harmonized guidelines for management of the pregnant Lassa fever patient are urgently needed to facilitate clinical decision-making and management of Lassa fever in West Africa, particularly in low-resource hard-to-reach environments. Health professionals working with pregnant women in endemic areas should be adequately trained and maintain a high index of suspicion, particularly during peak seasons.

There is currently no conclusive evidence on the efficacy of ribavirin in the management of Lassa fever in pregnancy and there has been no randomized controlled trial to assess the efficacy of ribavirin or other therapeutic agents in pregnant Lassa fever patients. This underscores the need for high-quality prospective studies and randomized controlled trials to assess the efficacy of different therapeutic agents in pregnant Lassa fever patients.

Most studies in our review did not report the gestational ages at which outcomes occurred and the outcome measures were rarely defined. As such we do not have sufficient data to synthesize evidence on pregnancy outcomes with respect to the trimester of pregnancy. There is an urgent need for a well-defined core set of outcomes for maternal Lassa infection to facilitate harmonization and evidence synthesis.

Our review has the following limitations. The meta-analyses consist of fewer than 10 studies with high amounts of heterogeneity in some of the estimates. However, we used the random effects models for the meta-analysis and a sensitivity analysis did not significantly impact the effect estimates. We attempted to explain the observed heterogeneity in a meta-regression and found that sample size, study design and year of outbreak did not explain the heterogeneity observed. Other possible confounders that may explain the heterogeneity observed include viral strains, trimester of pregnancy, the severity of illness and health system factors. However, we did not have sufficient data to analyze these. Another limitation is the moderate-to-high risk of bias in the included studies.

### Conclusions

In this review, we have shown that pregnancy is associated with a threefold increase in the risk of death from Lassa virus infection. While this underscores the need to prioritize pregnancy as a special subgroup of interest, major gaps in our understanding of the effects of Lassa fever in pregnancy remain and there is no conclusive evidence for the use of ribavirin for management of Lassa fever in pregnancy. Further research is needed to understand the clinical course of Lassa fever in pregnancy and identify pregnancy outcomes associated with Lassa fever and any associated protective, risk or prognostic factors. Research to validate diagnostics, therapeutics and preventive measures for Lassa fever in pregnancy need to be prioritized to facilitate clinical decision-making, guideline development and the design and implementation of prevention and control policies for Lassa fever in pregnancy.

## Supplementary Material

Figure_S1_traa011

Figure_S2_revised_traa011

Table_S1_revised_traa011

Table_S2_revised_20-01-2020_traa011

Table_S3_revised_20-01-2020_traa011

Supplementary_text_methods_Lassa_revised_20-01-2020_traa011
