# Supplementary material for: Lassa fever in pregnancy: a systematic review and meta-analysis
Source: Trans R Soc Trop Med Hyg. 2020 Mar 3;114(5):385–96. doi: 10.1093/trstmh/traa011 (PMC7197258; doi:10.1093/trstmh/traa011)
Supplement: Figure_S1_traa011 [file figure_s1_traa011.docx]

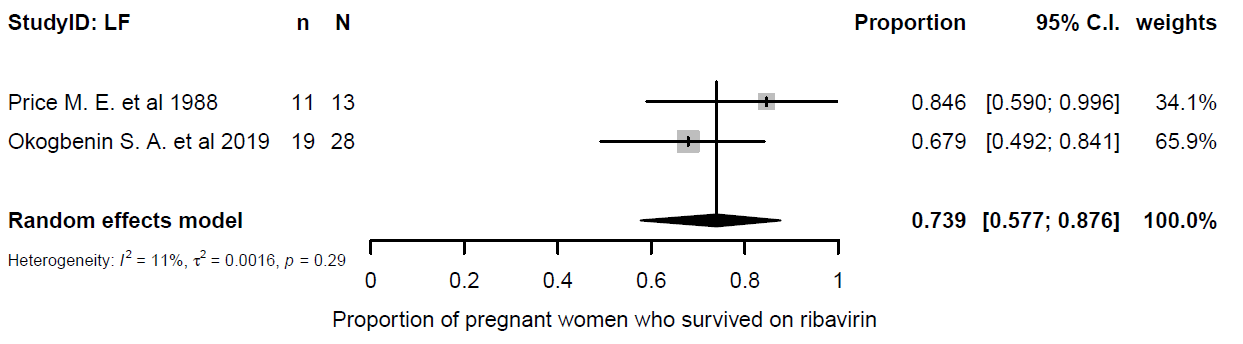


**Figure S1: Proportional meta-analysis forest plot of studies reporting survival of pregnant women with Lassa fever on ribavirin. I^2^=Higgins statistic, τ^2^=tau squared, P=p-value associated with Cochran’s Q for heterogeneity, ES- effect size, CI – confidence interval, LF-Lassa fever, n-number of pregnant women with symptom, N-total number of pregnant women in whom symptom was assessed.**
