# Supplementary material for: Lassa fever in pregnancy: a systematic review and meta-analysis
Source: Trans R Soc Trop Med Hyg. 2020 Mar 3;114(5):385–96. doi: 10.1093/trstmh/traa011 (PMC7197258; doi:10.1093/trstmh/traa011)
Supplement: Figure_S2_revised_traa011 [file figure_s2_revised_traa011.docx]

**Publication bias- Funnel plot asymmetry for studies reporting maternal death or foetal loss from Lassa fever during pregnancy.**


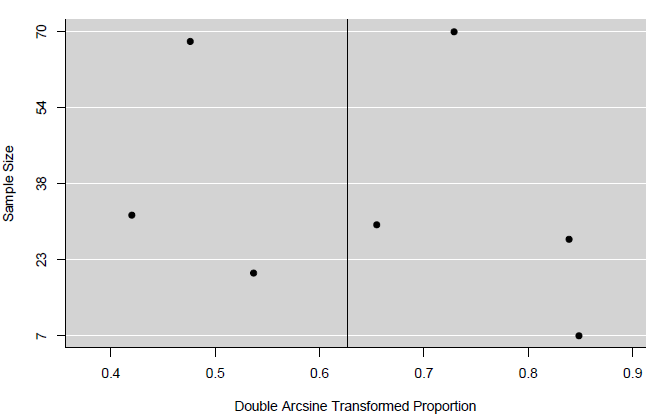

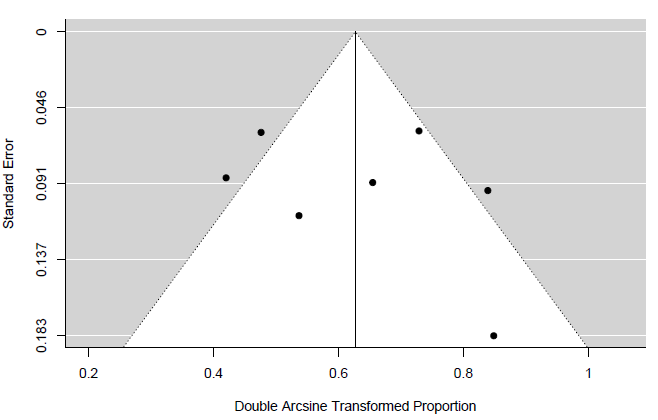


**B**

**A**


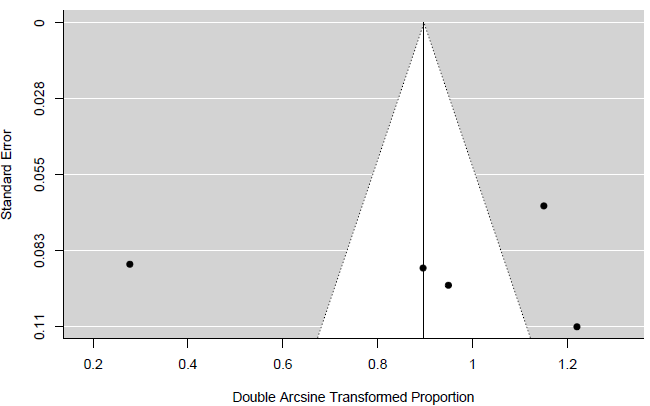

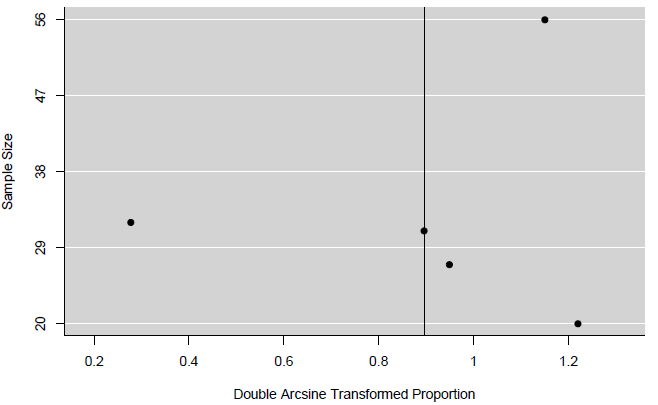


**D**

**C**


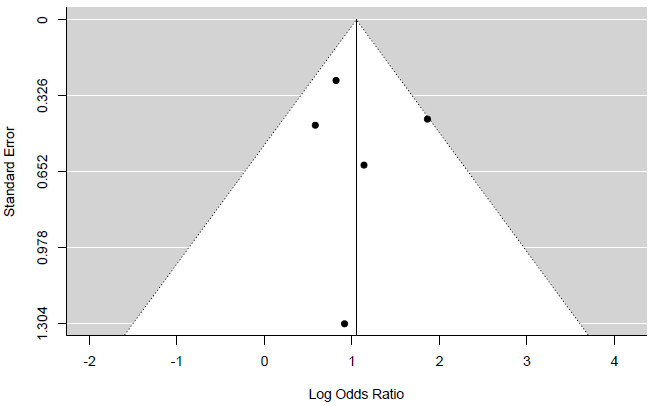

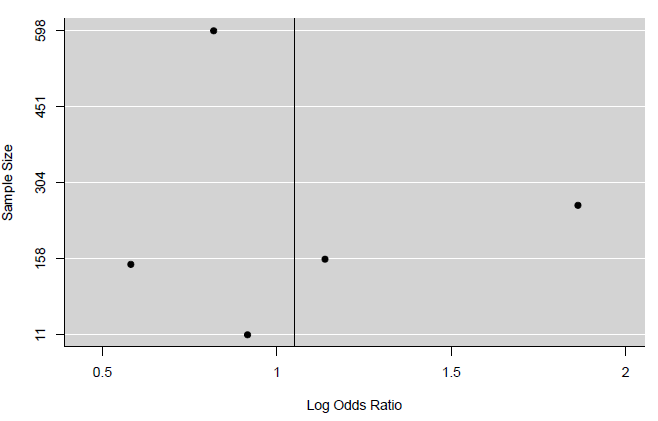


**F**

**E**

**Figure: Funnel plots assessing publication bias for studies included in the meta-analysis in Lassa fever**

1. Funnel plot of point estimates of the double-arcsine transformed maternal case fatality proportion with sample size as predictor
2. Funnel plot of point estimates of the double arcsine transformed maternal case fatality proportion with standard error as predictor
3. Funnel plot of point estimates of the double arcsine transformed foetal case fatality proportion with sample size as predictor
4. Funnel plot of point estimates of the double arcsine transformed foetal case fatality proportion with standard error as predictor
5. Funnel plot of point estimates of the log odds ratio for pregnant women with non-pregnant women with sample size as predictor
6. Funnel plot of point estimates of the log odds ratio for pregnant women with non-pregnant women with standard error as predictor.
