# Supplementary material for: Lassa fever in pregnancy: a systematic review and meta-analysis
Source: Trans R Soc Trop Med Hyg. 2020 Mar 3;114(5):385–96. doi: 10.1093/trstmh/traa011 (PMC7197258; doi:10.1093/trstmh/traa011)
Supplement: Table_S1_revised_traa011 [file table_s1_revised_traa011.docx]

**Table S1: Sample of some search terms used for the PubMed database**

| **Search** | **Search Terms** |
| --- | --- |
| #1 | Lassa fever AND ("preterm birth" or "premature birth" or prematur*) |
| #2 | Lassa fever AND "small for gestational age" |
| #3 | Lassa fever AND "low birth weight" |
| #4 | Lassa fever AND stillbirth |
| #5 | Lassa fever AND "neonatal death" |
| #6 | Lassa fever AND ("vertical transmission" or "suspected infection in newborn" or "confirmed infection in new born" or "mother to child transmission") |
| #7 | Lassa fever AND (pregnanc* or pregnant) AND (antiviral OR ribavirin OR favipiravir OR "management strategies" OR therap* OR management* OR treatment*) |
| #8 | Lassa fever AND ("postpartum haemorrhage" or "postpartum hemorrhage") |
| #9 | Lassa fever AND "preterm labor" |
| #10 | Lassa fever AND "preterm labour" |
| #11 | Lassa fever and "premature rupture of membranes" |
| #12 | Lassa fever and "spontaneous abortion" |
| #13 | Lassa fever AND (maternal mortality or neonatal mortality or perinatal mortality) |
| #14 | Lassa fever AND (maternal outcome* or pregnancy outcome* or pregnanc* or pregnant OR birth outcome* or perinatal outcome* or obstetric outcome* or fetal outcome* or antenatal or neonat* or foetal outcome*) |
| #15 | Lassa fever AND (pregnanc* or pregnant) AND (" infant feeding" or "breast feeding" or "complementary feeding" or "mixed feeding" or "bottle feeding") |
| #16 | Lassa fever AND (pregnanc* or pregnant) AND ( "caesarean delivery" or caesarean or "instrumental vaginal delivery" or "assisted vaginal delivery" or "operative delivery" or "spontaneous vaginal delivery" or "forceps delivery" or "vacuum delivery") |
| #17 | Lassa fever AND (pregnanc* or pregnant) AND ("medical termination of pregnancy" or abortion) |
| #18 | Lassa fever AND (pregnanc* or pregnant) AND ("blood products" or platelets or "whole blood") |
| #19 | Lassa fever AND (pregnanc* or pregnant) AND (immunetherapy or immunotherapy or steroid* or "immunosuppressive therapy" or "convalescent plasma") |
| #20 | Lassa* AND ("preterm birth" or "premature birth" or prematur*) |
| #21 | Lassa* AND "small for gestational age" |
| #22 | Lassa* AND "low birth weight" |
| #23 | Lassa* AND stillbirth |
| #24 | Lassa* AND "neonatal death" |
| #25 | Lassa* AND ("vertical transmission" or "suspected infection in newborn" or "confirmed infection in newborn" or "mother to child transmission") |
| #26 | Lassa* AND (pregnanc* or pregnant) AND (antiviral OR ribavirin OR favipiravir OR "management strategies" OR therap* OR management* OR treatment*) |
| #27 | Lassa* AND ("postpartum haemorrhage" or "postpartum hemorrhage") |
| #28 | Lassa* AND "preterm labor" |
| #29 | Lassa* AND "preterm labour" |
| #30 | Lassa fever and AND (congenital malformation or congenital abnormalit* or anomal*) |
| #31 | Lassa fever* AND (congenital malformation or congenital abnormalit* or anomal*) AND (congenital malformation or congenital abnormalit* or anomal*) |
| #32 | Lassa fever and AND ("intrauterine growth restriction" or "intrauterine growth retardation") AND ("intrauterine growth restriction" or "intrauterine growth retardation") |
| #33 | ((((Lassa fever* and pregnancy and ("clinical features" or "clinical characteristics" or " clinical presentation" or sign* or symptom*))) OR (Lassa fever and and pregnancy and ("clinical features" or "clinical characteristics" or " clinical presentation" or sign* or symptom*))) |
| #34 | (((((Lassa fever* and pregnant*)) OR (Lassa fever and and pregnant*)) |
