# Supplementary material for: Lassa fever in pregnancy: a systematic review and meta-analysis
Source: Trans R Soc Trop Med Hyg. 2020 Mar 3;114(5):385–96. doi: 10.1093/trstmh/traa011 (PMC7197258; doi:10.1093/trstmh/traa011)
Supplement: Table_S2_revised_20-01-2020_traa011 [file table_s2_revised_20-01-2020_traa011.docx]

**Table S2a: Murad et al Risk of Bias score - Adapted for Case Reports and Case Series Studies included in the Systematic Review of the Clinical epidemiology of viral hemorrhagic fevers in pregnancy**

| **Criteria** | **Decision rule** |
| --- | --- |
| **Selection** | Does the patient(s) represent(s) the whole experience of the investigator (center) or is the selection method unclear to the extent that other patients with similar presentation may not have been reported? |
| **Ascertainment** | Was the exposure adequately ascertained? |
|  | Was the outcome adequately ascertained? |
|  | Were other alternative causes that may explain the observation ruled out? |
|  | Was there a challenge/rechallenge phenomenon? |
|  | Was there a dose-response effect? |
| **Causality** | Was follow-up long enough for outcomes to occur? |
| **Reporting** | Is the case(s) described with sufficient details to allow other investigators to replicate the research or to allow practitioners to make inferences related to their own practice? |
| **Comment** |  |

Good - if 2 or more out of 3 for selection, causality and reporting and 3 or more out of ascertainment (if all fields applicable, if only 3 fields applicable then 2 or more of 3; Fair - if 1 or more out of 3 for selection, causality and reporting and 2 or more out of ascertainment (if all fields applicable, if only 3 fields applicable then 1 or more of 3) and poor - if none of the above criteria fulfilled. Maximum of 8 points.

**Table S2b: MODIFIED NEWCASTLE - OTTAWA QUALITY ASSESSMENT SCALE-Adapted for Cross-Sectional Studies included in the Systematic Review of the Clinical epidemiology of viral hemorrhagic fevers in pregnancy**

Note: A study can be awarded a maximum of one star for each numbered item within the Selection and Outcome categories. A maximum of one star can be given for Comparability

| **No.** | **Criterion** | | **Decision rule** | **Score**  **(*=1, no*=0)** |
| --- | --- | --- | --- | --- |
| SELECTION maximum of 4 points | | | | |
| 1 | Representativeness of sample | | 1. Selected or recruited from the same or similar populations (including the same time period)* 2. Not satisfying requirements in part (a), or not stated. 3. No description/unclear |  |
| 2 | Sample size: | | 1. All participants were included or at least 50% of eligible participants included.* 2. Not satisfying requirements in part (a), or not stated. 3. No description/unclear |  |
| 3 | Ascertainment of exposure | | 1. PCR or other laboratory confirmation of VHF conducted as part of study or found in medical records* 2. Probable 3. Suspected 4. Mixed methods for ascertainment of exposure 5. No description |  |
| 4 | Non-respondents | | 1. Response rate is at least 50% and there is satisfactory comparability between non-respondents and respondents. * 2. Comparability between respondents and non-respondents is unsatisfactory. 3. No description of the response rate or characteristics of the respondents and the non-respondents. |  |
| COMPARABILITY maximum 1 point | | | | |
| 1 | Comparability | | 1. Key potential confounding variables were measured and adjusted statistically for their impact on the relationship between exposure(s) and outcome(s)* 2. Study does not control for confounders 3. Not stated/ not determined/unknown/unclear |  |
| OUTCOME maximum 4 points | | | | |
| 1 | | General Assessment of outcome ^1^ | 1. Outcome was confirmed following observation by health care worker* 2. Outcome was extracted from medical records* 3. Patient-reported 4. No description of how outcome was ascertained |  |
| 2. | | Was gestational age accurately assessed? ^1^ | 1. Yes – determined by early Ultrasound - <14weeks* 2. Gestational age was determined by late ultrasound(≥14weeks), LNMP or neonatal assessment or a combination of methods 3. No description or unclear |  |
| 3 | | Follow-up | 1. Timeframe was sufficient so that one could reasonably expect to see an association between exposure and outcome if it existed and loss to follow-up was 20% or less * 2. Follow up rate <85% and no description of those lost provided 3. No statement |  |
| 4. | | Were the outcomes defined? | For outcomes which are not death.   1. All outcomes are clearly defined* 2. Only some outcomes are clearly defined 3. Not stated |  |

Quality assessment: Good - quality is all 9 points; Average - quality is 4 points in selection and 4 points in outcome, 2 -3 points in selection/outcome and 1 in comparability, 1 point in comparability and a total of 5 points; Poor - quality is <2 points in selection and outcome, 2 points in selection and outcome and 0 in comparability; If poor or average state why.

**Table S2c:** **MODIFIED NEWCASTLE - OTTAWA QUALITY ASSESSMENT SCALE-Adapted for Cohort Studies included in the Systematic Review of the Clinical epidemiology of viral hemorrhagic fevers in pregnancy**

Note: A study can be awarded a maximum of one star for each numbered item within the Selection and Outcome categories. A maximum of one star can be given for Comparability

| **No.** | **Criterion** | | **Decision rule** | **Score**  **(*=1, no*=0)** |
| --- | --- | --- | --- | --- |
| SELECTION maximum of 4 points | | | | |
| 1 | Representativeness of the exposed cohort | | 1. Consecutive eligible participants were selected, participants were randomly selected, or all participants were invited to participate from the source population* 2. Not satisfying requirements in part (a), or not stated. 3. No description/unclear |  |
| 2 | Selection of the non-exposed cohort | | 1. Selected from the same source population*   *that is the VHF negative patients were selected from the same population as the VHF positive ones*   1. Selected from a different source population 2. No description |  |
| 3 | Ascertainment of exposure | | 1. PCR or other laboratory confirmation of VHF conducted as part of study or found in medical records* 2. Probable 3. Suspected 4. Mixed methods for ascertainment of exposure 5. d) No description |  |
| 4 | Demonstration that outcome of interest was not present at study start | | 1. Yes (paper clearly states that outcome(s) was not present)* 2. This was only indicated for some outcomes 3. No 4. Unclear |  |
| COMPARABILITY maximum 1 point | | | | |
| 1 | Comparability of cohorts on the basis of the design or analysis | | 1. study controls for confounders and methods used are described* 2. study controls for confounders but no methods described 3. study does not control for confounders 4. not stated/ not determined/unknown/unclear   *Note: Exposed and non-exposed individuals must be matched in the design and/or confounders must be adjusted for in the analysis. Alone statements of no differences between groups or that differences were not statistically significant are not sufficient.* |  |
| OUTCOME maximum 4 points | | | | |
| 1 | | General Assessment of outcome ^1^ | 1. Outcome was confirmed following observation by health care worker* 2. Outcome was extracted from medical records* 3. Patient-reported 4. No description of how outcome was ascertained |  |
| 2. | | Was gestational age accurately assessed? ^1^ | 1. Yes – determined by early Ultrasound - <14 weeks* 2. Gestational age was determined by late Ultrasound (≥14 weeks), LNMP or neonatal assessment or a combination of methods 3. No description or unclear |  |
| 3 | | Adequacy of follow up of cohorts | 1. Complete follow-up – all participants accounted for* 2. Subjects lost to follow up unlikely to introduce bias (<15% lost to follow up, or description provided of those lost*) 3. Follow up rate <85% and no description of those lost provided 4. No statement |  |
| 4. | | Were the outcomes defined? | For outcomes which are not death.   1. All outcomes are clearly defined* 2. Only some outcomes are clearly defined 3. Not stated |  |

Quality assessment: Good - quality is all 9 points; Average - quality is 4 points in selection and 4 points in outcome, 2 -3 points in selection/outcome and 1 in comparability, 1 point in comparability and a total of 5 points; Poor - quality is <2 points in selection and outcome, 2 points in selection and outcome and 0 in comparability; If poor or average state why.

1. Wedi COO, Kirtley S, Hopewell S, et al. Perinatal outcomes associated with maternal HIV infection: a systematic review and meta-analysis. *The lancet HIV* 2016; **3**(1): e33-e48.
