# Supplementary material for: Lassa fever in pregnancy: a systematic review and meta-analysis
Source: Trans R Soc Trop Med Hyg. 2020 Mar 3;114(5):385–96. doi: 10.1093/trstmh/traa011 (PMC7197258; doi:10.1093/trstmh/traa011)
Supplement: Table_S3_revised_20-01-2020_traa011 [file table_s3_revised_20-01-2020_traa011.docx]

**Table S3a: Criteria used for the identification of research gaps**

| Gap category | Reason for gap | Description |
| --- | --- | --- |
| A | Insufficient or no information ^1^ | No studies describing this outcome; insufficient literature or number of studies describing this outcome ^1^; or not enough studies to estimate a summary effect (perform a meta-analysis) ^1^ |
| B | Imprecise information ^1^ | A meta-analysis was done but, small sample sizes or few studies included in the meta-analysis or there is a high amount of heterogeneity or extremely wide confidence intervals ^1^ |
| C | Biased information ^1^ | The aggregated risk of bias score for studies in the meta-analysis is high, or publication bias ^1^ |
| D | Not the right information ^1^ | The information provided in the studies is not on the outcomes we were interested in or is mostly from case reports or the follow-up time is too short to observe the effect reported ^1^. |
| E | Inconsistent information ^1^ | The direction of effect in the different studies included in the meta-analysis varies or large differences in effect sizes ^1^ |

1. Robinson KA, Saldanha IJ, McKoy NA. Development of a framework to identify research gaps from systematic reviews. *J Clin Epidemiol* 2011; **64**(12): 1325-30.

**Table S3b: Gap analysis expected outcomes based on research objectives on a systematic review of clinical epidemiology of the viral hemorrhagic fevers in pregnancy.**

| Research Question/Objective | Outcomes |
| --- | --- |
| To summarise and critically appraise evidence on the clinical characteristics or presentation of viral hemorrhagic fevers in pregnant women reported in the peer-reviewed and grey literature. | Clinical features of viral hemorrhagic fevers in pregnant women  Gestational age and estimation methods  The clinical course of viral hemorrhagic fevers in pregnant women  Coinfections and their effect on course of viral hemorrhagic fevers  Comorbidities and their effect on course of viral hemorrhagic fevers |
| To summarise and critically appraise evidence on the reported maternal outcomes of viral hemorrhagic fevers during pregnancy reported in the peer-reviewed and grey literature. | Maternal death  Postpartum hemorrhage  Premature (prelabour) rupture of membranes (pre-term or term)  Preterm labor  Complications (Hemolysis, Elevated Liver enzymes and Low Platelet count syndrome, pre-eclampsia, ocular manifestations, hepatitis, antepartum hemorrhage, diabetes, hepatic insufficiency or failure, Disseminated Intravascular Coagulopathy (DIC), Acute respiratory distress syndrome, myocarditis, intracranial bleeding) |
| To summarise and critically appraise evidence on the reported fetal and neonatal outcomes of maternal infection with viral hemorrhagic fevers reported in the peer-reviewed and grey literature. | Miscarriage  Intrauterine fetal demise/death  Stillbirths  Neonatal death  Prematurity  Small-for-gestational-age and intrauterine growth restriction/retardation  Low birthweight  Vertical transmission or Mother-to-Child-transmission of Lassa fever and  Birth defects  Clinical features and complications in new-born (necrotizing enterocolitis, Apgar score below 7 in first 5 minutes, DIC, respiratory failure, hemorrhage, coma) |
| To summarise and critically appraise evidence on the reported clinical management practices aimed at improving pregnancy outcomes of viral hemorrhagic fevers during pregnancy reported in the peer-reviewed and grey literature. | Medical practices for management of pregnant women with viral hemorrhagic fevers  Obstetric management of pregnant women with viral hemorrhagic fevers  Effectiveness of different treatments  Management of neonates born to mothers with viral hemorrhagic fevers during pregnancy  Choice of infant feeding practices |
