# Supplementary material for: Lassa fever in pregnancy: a systematic review and meta-analysis
Source: Trans R Soc Trop Med Hyg. 2020 Mar 3;114(5):385–96. doi: 10.1093/trstmh/traa011 (PMC7197258; doi:10.1093/trstmh/traa011)
Supplement: Supplementary_text_methods_Lassa_revised_20-01-2020_traa011 [file supplementary_text_methods_lassa_revised_20-01-2020_traa011.docx]

**Supplementary text**

**Clinical epidemiology of Lassa fever in pregnancy: A systematic review and meta-analysis.**

PROSPERO registration: CRD42018097022.

**Methods:**

**Search strategy and eligibility criteria**

The following bibliographic databases were searched from their respective inception dates until June 25^th^, 2018: PubMed, Web of Science, EMBASE (Excerpta Medica dataBASE), the World Health Organization Global Health Library (WHOGL) and CINAHL (Cumulative Index to Nursing and Allied Health Literature). Additionally, clinical trial databases such as Cochrane Central Register of Controlled Trials, the Cochrane Pregnancy and Childbirth Register, the Cochrane Infectious Diseases Register, the ISRCTN Registry (International Standard Randomised Controlled Trial Number Registry), WHO International Clinical Trials Registry Platform, the European (EU) Clinical Trials Register, the Pan African Clinical Trials Registry and the ClinicalTrials.gov database were searched. There were no language or date restrictions.

KND developed the search strategy in consultation with a librarian (Nia Roberts). A combination of MeSH terms and keywords were used to capture pregnancy and the selected viral hemorrhagic fevers for both published and unpublished (grey) literature (supplementary Table S1). Variations of these terms were used to search the other databases according to the level of term indexation for each database. The references from relevant reviews and included studies were also searched for additional citations. A repeat search of the databases was conducted on **September 30^th^, 2019** to identify more recent publications.

**Selection of studies**

**Inclusion criteria**

Studies were included if they presented original data regarding the selected viral hemorrhagic fevers in pregnant women. The VHFs of interest were Lassa fever, Ebola and Marburg virus disease, Rift Valley fever, and Crimean-Congo hemorrhagic fever. Exposure was defined by clinical or laboratory criteria. Outcomes were broadly classified as clinical characteristics, maternal and perinatal outcomes or complications and management practices related to the viral hemorrhagic fevers and pregnancy that were reported in the literature. Outcomes were not predefined for the review and definitions were extracted from each citation.

All study designs such as case series studies, comparative studies (cross-sectional, cohort or case-control studies) and experimental studies (e.g. randomized trials, non-randomised controlled trials) were considered for inclusion. Additionally, original reports, briefs, letters, editorials, correspondence, and comments were considered for inclusion. This inclusive approach was adopted because viral hemorrhagic fevers are rare and the population of interest (pregnant women) is generally excluded from trials and other analytical studies, as such it was expected that most of the papers will be case series and case reports.

**Data extraction**

For the purpose of this review, a case report was a study describing a single patient. ^1^ Dekker’s criteria were used to differentiate a case series from a cohort study. ^2^ According to Dekker, a case series is a study, which looks at the participants based on outcome regardless of exposure or a study, which sampled participants on the basis of specific exposure and a specific outcome ^2,3^. A cohort is a study where patients are sampled on the basis of exposure and followed up for the occurrence of an outcome over a specified follow-up period; additionally, a study can be a cohort if the available information allows for an exposure - based comparison. ^2,3^ All surveys or surveillance studies were classified as cross-sectional studies.

A standardized, pre-piloted form was used to extract data from the included studies and a list of data extracted from each paper is indicated in the box below. The citations were screened by a group of five reviewers (NDK, CA, CB, SB, and MT) using an online systematic review software program – Rayyan. ^4^ The titles and abstracts of each citation were independently screened by two reviewers. The full texts were then retrieved and independently assessed for eligibility by two reviewers. Any disagreements between the reviewers over the eligibility of any particular study was resolved through discussion with a third reviewer.

Data were extracted from each citation by two reviewers independently and any discrepancies were resolved through discussion with a third author where necessary. Where a feature or outcome of interest was not listed, the reviewers indicated this as not stated.

**Box 1: Type of data extracted from the articles**

| The data extracted from the included articles were first author, year of publication, enrolment period, study design, direction of the study, total number of participants in the study before analysis, total number of participants in the study included in analysis, total number of cases in the study, total number of cases included in analysis, case definition, number of pregnant women who were positive included in analysis, number of pregnant women who were negative, total number of controls in the study, definition of controls, total number of controls included in analysis, method used to make diagnosis, test used for laboratory diagnosis, gestational age (GA) estimation methods, methods used to correct for confounding, patients lost-to-follow-up (LFTU), missing data, number of pregnant women excluded from analysis and reason, Gestational age at admission, ages of pregnant women, history of contact with human suspected of the VHF under study, history of contact with animal reservoir, coinfections, comorbidities, clinical features on and during admission (number with clinical feature, total number in who clinical features were reported), length of hospital stay, duration of illness, clinical course during hospitalization e.g. improved then discharged, readmitted-why when improved following delivery, deteriorated following delivery etc, maternal outcomes such as death, postpartum hemorrhage etc, perinatal outcomes such as miscarriage or abortion, stillbirth, live births, neonatal death etc, definition of each outcome, clinical management practices (number of patients who received management described, outcome as a result of management strategy, effect sizes for outcomes and management practices). |
| --- |

**Risk of bias (quality) assessment**

Two review authors independently assessed the risk of bias in included studies, using pre-existing tools appropriate to the study design. Where discordance in ranking occurred this was resolved by consensus or by discussion with a third reviewer. Although we provided aggregated scores, the risk of bias was colour-coded to allow for a better interpretation of the study quality by readers given that an aggregated score fails to highlight where specific weaknesses in the reported study design are found. ^5^

The risk of bias (quality) assessment tools were adapted to suit the pathogens being addressed in this review because VHFs have a short incubation and follow up period, we expected that the follow up period to observe outcomes was much shorter than that indicated in pre-existing tools, for example, the Newcastle-Ottawa scale which requires a follow up of three months. Additionally, our population of interest is pregnant women as such gestational ages and estimation methods are necessary to ensure methodological robustness.

Case reports and case series studies were evaluated using a tool developed by Murad M. H.and colleagues ^6^ (supplementary Table S2), which evaluates studies on the basis of four domains similar to those in the Newcastle-Ottawa scale for cohorts and case-control studies. The tool has questions based on eight criteria to which we organized four categories of answers, ‘yes’, ‘no’, ‘unclear/unsure’ and ‘not applicable’. Studies for which the criteria were irrelevant were labelled ‘not applicable’ and ‘unclear or unsure’ if the information was missing or not reported in the study. We chose the Murad tool over the CARE criteria for case reports,^7^and the NIH criteria for case series studies,^8^ because the CARE tool was not developed for evaluation of methodological quality but as a checklist to ensure complete reporting ^7^ for case reports.

The modified Newcastle-Ottawa scale was used to evaluate the risk of bias in cohort-type studies.^9^ It comprises lead questions which assess three domains – selection, comparability, and outcome. The scale was modified to include another outcome criteria which was specific to pregnant women (gestational age estimation) ^10^ and definition of outcomes were included in the assessment. The scale uses a star system, where each question or criteria can receive a maximum of one star only (supplementary Table S2).

**Statistical analysis**

The gestational age at which perinatal outcomes occurred was generally not reported in the studies. As such, for this review, we defined fetal loss as any death occurring before or during labour and delivery, including miscarriages, intrauterine fetal death, and stillbirths. Maternal death was defined as the death of the pregnant woman during pregnancy, labour, and delivery and within one month after delivery. Neonatal death was defined as the death of a new-born within one month of birth.

We extracted dichotomous data from the studies to generate proportions or odds ratios depending on the available information irrespective of study quality score. The three most commonly reported outcomes (maternal death, fetal loss, and neonatal death) were summarised in meta-analysis forest plots. We only performed a meta-analysis when two or more studies reported on the same outcome and included at least five pregnant women or live births.

The fetal case fatality proportion was calculated as the number of fetal losses divided by the total number of fetuses in each study. The maternal case fatality proportion was calculated as the number of maternal deaths divided by the total number of pregnant women in each study and the neonatal case fatality proportion was calculated as the number of new-born deaths divided by the total number of live births in each study.

Where the information was available, we calculated the odds ratios (OR) afresh by comparing the odds of death in pregnant Lassa fever positive women to that in non-pregnant Lassa positive women.

All statistical analyses were done using R Statistical Software version 3.6.1.^11-13^ We used the *metaprop* command for the proportional meta-analysis and the *metabin* command was used to summarise the effect estimate for odds ratios because they implement procedures specific to binary outcome data. The weighted summary proportion for the proportional meta-analysis was calculated using the Freeman-Tuckey double arcsine transformation, ^14,15^ which converts data using an arcsine square root transformation, is well-suited to binomial data with extreme proportions close to 0% and 100%, and stabilizes variances in proportions. ^13-15^ Box 2 provides an example of some of the code used to develop the forest plots.

**Box 2: Example of code used for proportional meta-analysis of maternal case fatality in R**

| datlm=read.csv("lfmm.csv", header=T, sep=",")  > ies.da=escalc(xi=deathpw, ni=samplepw, data=datlm, measure="PFT", add=0)  > pes.da=rma(yi, vi, data=ies.da, method="DL", weighted=TRUE)  > pes=predict(pes.da, transf=transf.ipft.hm, targ=list(ni=datlm$samplepw))  > pes.forestlm=metaprop(deathpw, samplepw, studyname, dat=datlm, sm="PFT", method.ci="NAsm", method.tau = "DL")  >  > forest(pes.forestlm, xlim = c(0,1), pscale = 1, rightcols = c("effect", "ci", "w.random"), rightlabs = c("Proportion", "95% C.I.", "weights"), leftcols = c("studlab","deathpw", "samplepw"), leftlabs = c("StudyID: LF", "n", "N"), xlab = "Maternal Case Fatality",fs.xlab = 8, fs.study = 8,fs.study.labels = 8, fs.heading = 8, squaresize = 0.5, col.square = "black",col.square.lines = "black", col.diamond = "navy", col.diamond.lines = "navy", comb.fixed = FALSE, lty.fixed = 0, lty.random = 1, type.study = "square", type.random = "diamond", ff.fixed = "italic", ff.random= "bold", fs.hetstat=6, fs.random= 8, fs.axis=8, print.tau2 = TRUE, print.Q = FALSE, print.pval.Q = TRUE, print.I2 = TRUE, digits= 3) |
| --- |

A random-effects model was used to calculate a weighted summary estimate and the 95% confidence interval (CI) for the association between viral hemorrhagic fevers and maternal death, fetal loss or neonatal death. We estimated the following parameters: Cochran’s Q which looks at differences in true effect sizes, tau squared (τ^2^) which is an estimate of the true variance of effect sizes between studies and Higgins I^2^ which is an estimate of the proportion of the observed variance that reflects real differences in effect sizes. For our study, the degree of heterogeneity was interpreted as none (<25%), low (25-49%), moderate (50-74%), or high (≥ 75%).^16^ We evaluated the reasons for the observed variance only if I^2^ was over 50%. Studies were included in the meta-analysis if they included 10 or more pregnant women or live births. A meta-regression was performed to assess the effect of study design (cohort or other design), and year of the outbreak (before or after 2000) on summary estimates.

Peter’s test was used in combination with a funnel plot to assess potential publication bias.^17^ Funnel plots were derived using a mixed-effects model with sample size as the predictor and the double arcsine transformed proportion. Peter’s test was chosen over Egger’s test^18^ because for proportional meta-analysis particularly those with proportions at either extreme (0% and 100%), the funnel plots with standard error as predictor tend to overestimate asymmetry and sample size is considered a better predictor. ^19^ Additionally, Peter’s test is more stable than Egger’s test when there is high between-study heterogeneity. ^17^ Begg’s correlation was not used because the rank correlation test has low power when the number of studies is below twenty-five. ^20^

**Gap analysis**

The identification of research gaps in health sciences is often done arbitrarily and no validated systematic method to identify research gaps exist to the best of our knowledge. We adapted a framework developed by Robinson and colleagues^21^ for identifying research gaps from literature reviews. The framework allows not only for identification of gaps but the reasons for the gaps. The gaps are classified under 4 categories namely: A. Insufficient or imprecise information; B. Biased information; C. Inconsistency or unknown consistency; and D. Not the right information. All research gaps were framed based on a specific population, intervention, comparator, and outcomes.

Given that our research questions did not address any specific interventions we modified the framework to focus on population, exposure, and outcomes. The viral hemorrhagic fevers are rare diseases and our selected population (pregnant women) are rarely included in clinical research, ^22^ as such we expected that a meta-analysis will not be possible for all outcomes. In our opinion, it is important to assess the evidence based on the meta-analysis separately, as such where Robinson and colleagues ^21^ discuss gaps based on four categories described in the preceding paragraph, we had five categories by separating the options for insufficient and imprecise information into two distinct categories (supplementary Table 3). For each objective, we presumed certain outcomes should be reported and as such assessed the evidence or lack thereof based on these objectives (supplementary Table S3).

# **References**

1. Rison RA. A guide to writing case reports for the Journal of Medical Case Reports and BioMed Central Research Notes. *J Med Case Rep* 2013; **7**(1): 239.

2. Dekkers OM, Egger M, Altman DG, et al. Distinguishing case series from cohort studies. *Ann Intern Med* 2012; **156**(1_Part_1): 37-40.

3. Mathes T, Pieper D. Clarifying the distinction between case series and cohort studies in systematic reviews of comparative studies: potential impact on body of evidence and workload. *BMC Med Res Methodol* 2017; **17**(1): 107.

4. Ouzzani M, Hammady H, Fedorowicz Z, et al. Rayyan---a web and mobile app for systematic reviews. *Syst Rev* 2016; **5**(1): 210.

5. Viswanathan M, Ansari MT, Berkman ND, et al. Assessing the risk of bias of individual studies in systematic reviews of health care interventions. Agency for Healthcare Research and Quality Methods Guide for Comparative Effectiveness Reviews AHRQ 2012.

6. Murad MH, Sultan S, Haffar S, et al. Methodological quality and synthesis of case series and case reports. *BMJ Evid Based Med* 2018; **23**(2): 60-3.

7. Gagnier JJ, Kienle G, Altman DG, et al. The CARE guidelines: consensus-based clinical case reporting guideline development. *BMJ Case Rep* 2013; **2013**: bcr2013201554.

8. US Department of Health & Human Services - National Heart LaBI. Quality Assessment Tool for Case Series Studies. 2014. <https://www.nhlbi.nih.gov/health-topics/study-quality-assessment-tools> (accessed 12 September 2018).

9. Wells G, Shea B, O’connell D, et al. The Newcastle-Ottawa Scale (NOS) for assessing the quality of nonrandomised studies in meta-analyses. Ottawa (ON): Ottawa Hospital Research Institute; 2009. <http://www.ohri.ca/programs/clinical_epidemiology/oxford.asp> (accessed 20 December 2017).

10. Wedi COO, Kirtley S, Hopewell S, et al. Perinatal outcomes associated with maternal HIV infection: a systematic review and meta-analysis. *The lancet HIV* 2016; **3**(1): e33-e48.

11. RDevelopmentCoreTeam. R: A language and environment for statistical computing. Vienna, Austria: R Foundation for Statistical Computing.; 2008.

12. Viechtbauer W. Conducting Meta-Analyses in R with the metafor Package. *J Stat Softw* 2010; **36**(3): 1-48.

13. Wang N. How to Conduct a Meta-Analysis of Proportions in R: A Comprehensive Tutorial. 2018. dx.doi.org/10.13140/RG.2.2.27199.00161 (accessed 30 September 2018).

14. Freeman MF, Tukey JW. Transformations Related to the Angular and the Square Root. *Annals of mathematical statistics* 1950; **21**(4): 607-11.

15. Miller JJ. The Inverse of the Freeman – Tukey Double Arcsine Transformation. *Am Stat* 1978; **32**(4): 138.

16. Higgins JPT, Green S. Cochrane handbook for systematic reviews of interventions: John Wiley & Sons; 2011.

17. Peters JL, Sutton AJ, Jones DR, et al. Comparison of two methods to detect publication bias in meta-analysis. *JAMA* 2006; **295**(6): 676-80.

18. Egger M, Smith GD, Schneider M, et al. Bias in meta-analysis detected by a simple, graphical test. *BMJ* 1997; **315**(7109): 629.

19. Hunter JP, Saratzis A, Sutton AJ, et al. In meta-analyses of proportion studies, funnel plots were found to be an inaccurate method of assessing publication bias. *J Clin Epidemiol* 2014; **67**(8): 897-903.

20. Begg CB, Mazumdar M. Operating Characteristics of a Rank Correlation Test for Publication Bias. *Biometrics* 1994; **50**(4): 1088-101.

21. Robinson KA, Saldanha IJ, McKoy NA. Development of a framework to identify research gaps from systematic reviews. *J Clin Epidemiol* 2011; **64**(12): 1325-30.

22. Gomes MF, de la Fuente-Núñez V, Saxena A, et al. Protected to death: systematic exclusion of pregnant women from Ebola virus disease trials. *Reprod Health* 2017; **14**(Suppl 3): 172.
